# Tris(1,10-phenanthroline-κ^2^ *N*,*N*′)iron(II) bis­(1,1,3,3-tetra­cyano-2-eth­oxy­propenide) hemihydrate

**DOI:** 10.1107/S1600536812048611

**Published:** 2012-12-05

**Authors:** Zouaoui Setifi, Fatima Setifi, Seik Weng Ng, Abdelghani Oudahmane, Malika El-Ghozzi, Daniel Avignant

**Affiliations:** a Département de Technologie, Faculté de Technologie, Université 20 Août 1955 de Skikda, 21000 Skikda, Algeria; b Unité de Recherche de Chimie de l’Environnement et Moléculaire Structurale (CHEMS), Université Mentouri Constantine, 25000 Constantine, Algeria; c Laboatoire de Chimie, Ingénierie Moléculaire et Nanostructures (LCIMN), Université Ferhat Abbas de Sétif, 19000 Sétif, Algeria; d Department of Chemistry, University of Malaya, 50603 Kuala Lumpur, Malaysia; e Chemistry Department, King Abdulaziz University, PO Box 80203 Jeddah, Saudi Arabia; f Clermont Université, Université Blaise Pascal, Institut de Chimie de Clermont-Ferrand, BP 10448, 63000 Clermont-Ferrand, France; g CNRS UMR 6296, ICCF, BP 80026, 63171 Aubière, France

## Abstract

In the title hydrated mol­ecular salt, [Fe(C_12_H_8_N_2_)_3_](C_9_H_5_N_4_O)_2_·0.5H_2_O, the water mol­ecule site is half-occupied. The Fe—N bond lengths within the octa­hedral tris-chelate [Fe(phen)3]^2+^ ion (phen is 1,10-phenantroline) are indicative of a low-spin *d* ^6^ electronic configuration for the metal ion. The C—N, C—C and C—O bond lengths in the polynitrile anions indicate extensive electronic delocalization. In the crystal, the components are linked through O—H⋯N hydrogen bonds, forming [100] chains, as well as through Coulombic inter­actions.

## Related literature

For background to 1,10-phenanthroline as a chelating ligand, see: Hoshina *et al.* (2000 ▶); Hwang & Ha (2006 ▶); Aparici Plaza *et al.* (2007 ▶); Zhou & Guo (2007 ▶). For a related structure, see: Cai & Zhan (2012 ▶). For further synthetic details, see: Middleton & Engelhardt (1958 ▶).
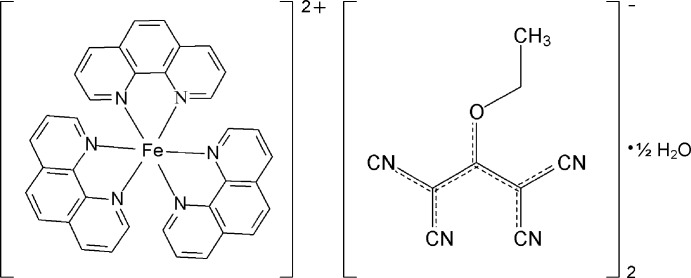


## Experimental

### 

#### Crystal data

[Fe(C_12_H_8_N_2_)_3_](C_9_H_5_N_4_O)_2_·0.5H_2_O
*M* *_r_* = 975.81
Triclinic, 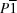

*a* = 9.3497 (3) Å
*b* = 14.1736 (4) Å
*c* = 18.6086 (6) Å
α = 94.462 (2)°
β = 96.562 (1)°
γ = 101.129 (1)°
*V* = 2391.12 (13) Å^3^
*Z* = 2
Mo *K*α radiation
μ = 0.38 mm^−1^
*T* = 293 K
0.55 × 0.35 × 0.15 mm

#### Data collection

Bruker SMART APEX CCD diffractometer
Absorption correction: multi-scan (*SADABS*; Sheldrick, 1996 ▶) *T*_min_ = 0.820, *T*_max_ = 0.946
38167 measured reflections
10845 independent reflections
6909 reflections with *I* > 2σ(*I*)
*R*_int_ = 0.043

#### Refinement

*R*[*F* ^2^ > 2σ(*F* ^2^)] = 0.049
*wR*(*F* ^2^) = 0.134
*S* = 1.02
10845 reflections
661 parameters
40 restraints
H-atom parameters constrained
Δρ_max_ = 0.36 e Å^−3^
Δρ_min_ = −0.43 e Å^−3^

### 

Data collection: *APEX2* (Bruker, 2008 ▶); cell refinement: *SAINT* (Bruker, 2008 ▶); data reduction: *SAINT*; program(s) used to solve structure: *SHELXS97* (Sheldrick, 2008 ▶); program(s) used to refine structure: *SHELXL97* (Sheldrick, 2008 ▶); molecular graphics: *X-SEED* (Barbour, 2001 ▶) and *Mercury* (Macrae *et al.*, 2006 ▶); software used to prepare material for publication: *publCIF* (Westrip, 2010 ▶).

## Supplementary Material

Crystal structure: contains datablock(s) global, I. DOI: 10.1107/S1600536812048611/hb6994sup1.cif

Structure factors: contains datablock(s) I. DOI: 10.1107/S1600536812048611/hb6994Isup2.hkl

Additional supplementary materials:  crystallographic information; 3D view; checkCIF report

## Figures and Tables

**Table 1:** Selected bond lengths (Å)

Fe1—N6	1.9563 (19)
Fe1—N5	1.9654 (18)
Fe1—N4	1.9686 (19)
Fe1—N1	1.9752 (18)
Fe1—N2	1.9819 (18)
Fe1—N3	1.9836 (18)

**Table 2:** Hydrogen-bond geometry (Å, °)

*D*—H⋯*A*	*D*—H	H⋯*A*	*D*⋯*A*	*D*—H⋯*A*
O1w—H11⋯N7	0.84	2.17	2.996 (5)	169
O1w—H12⋯N8^i^	0.84	2.25	3.078 (5)	169

Symmetry code: (i) 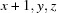.

**Fig. 1.**
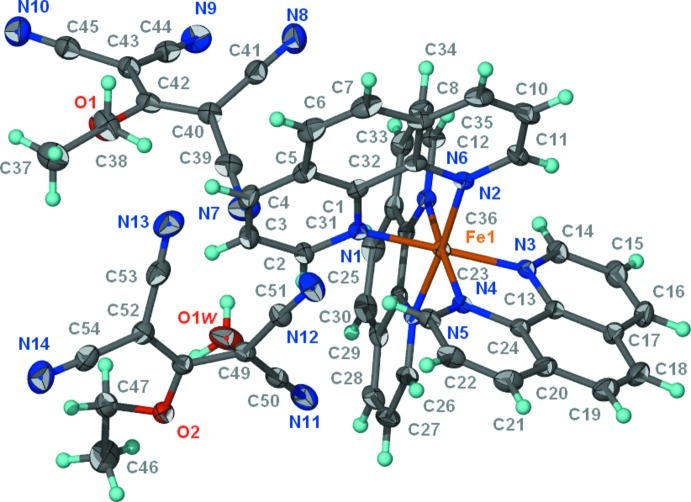
The molecular structure of (I) at the 30% probability level; hydrogen atoms are drawn as spheres of arbitrary radius. The disorder in the ethyl groups is not shown.

**Fig. 2.**
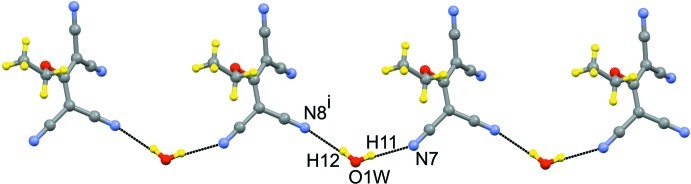
A View of the one-dimensional {[(H2O)(tcnoet)2]2-}n anion chain in (I). O—H···N Hydrogen bonds are represented by dashed lines. [details are given in Table 1; symmetry code: (i) x + 1, y, z.]

## supplementary crystallographic information

### Comment

1,10-Phenanthroline (phen) is a widely utilized chelating ligand in coordination chemistry and a lot of complexes with phen as a ligand have been reported (Hoshina *et al.*, 2000; Hwang & Ha, 2006; Zhou & Guo, 2007). Recently, Cai *et al.* (2012) has reported the structure of a complex with [Fe(phen)_3_]^2+^ as cation and 1,1-dicyano-2-ethoxy-2-oxoethanide as counter-ion. We report here the synthesis and crystal structure of the title compound, in which a polynitrile anion acts as counter-ion.

The structure of (I) is composed of a discrete [Fe(phen)_3_]^2+^ cations, uncoordinated tcnoet anions and water molecules of crystallization (Fig. 1).

Six nitrogen atoms from three bidentate phen ligands form a distorted octahedron around the iron atom with a mean Fe1—N bond length of 1.972 (18) Å. The main distortion from the octahedral geometry is observed in the values of the angles subtended by phen at the metal atom (82.36 (7)°, 82.50 (8)° and 83.02 (8)° for N1—Fe1—N2, N4—Fe1—N3 and N6—Fe1—N5, respectively) which deviate significantly from the ideal value of 90°. Each phenanthroline ligand is coplanar to within 0.04 Å. The average value of dihedral angle between pairs of phenanthroline planes is 82,1(3)°. The carbon-carbon and carbon-nitrogen intra-ring bond lengths agree with those observed in other metal complexes with chelating phen (Hoshina *et al.*, 2000; Aparici Plaza *et al.*, 2007; Cai & Zhan, 2012).

Examination of the intermolecular contacts in the crystal structure of (I) reveals that the main contacts are associated with O—H···N hydrogen bonds involving the water H atoms and those of the N atoms of the CN groups of the tcnoet anions containing atoms O1 (Table 1). In the crystal, this leads to the formation of an infinite one-dimensional {[(H_2_O)(tcnoet)_2_]^2-^}_n_ anionic chains (Fig. 2), which interact with the cationic entities [Fe(phen)_3_]^2+^ and the tcnoet anions involving atoms O2 *via* coulombic forces.

Due to the presence of the supplementary π electron systems of the cyano groups, the tcnoet ligands of (I) present a strong electronic delocalization, as indicated by C—N, C—C and C—O bond lengths.

### Experimental

Potassium 1,1,3,3-tetracyano-2-ethoxypropenide (Ktcnoet) was prepared by reaction in ethanol of 1,1-diethoxy-2,2-dicyanoethene with malononitrile and potassium t-butoxide as described in Ref (Middleton & Engelhardt, 1958). Under aerobic conditions, an aqueous solution of Fe(BF_4_)_2_.6H_2_O (0.034 g, 5 ml) was slowly added to an ethanolique solution of 1,10-phenanthroline (0.020 g, 5 ml). To the resulting red soluion was added dropwise an aqueous solution of the polynitrile potassium salt Ktcnoet (0.045 g, 10 ml). The final solution was filtered and the filtrate was allowed to evaporate for few days at r.t. offorded red prisms of (I).

### Refinement

Carbon-bound H-atoms were placed in calculated positions (C—H 0.93 to 0.97 Å) and were included in the refinement in the riding model approximation, with U(H) set to 1.2 to 1.5 U(C).

The water H-atoms were placed in calculated positions on the basis of hydrogen bonding and their temperature factors tied by a factor of 1.5 times. As the oxygen atom displayed extremely large temperature factors, the refinement of its occupancy was attempted. This refined to nearly 0.5; the occupancy was then set to exactly 0.5.

The two ethyl groups are disordered over two positions in a 1:1 ratio. The C—C distance was restrained to 1.54±0.01 Å. For each group, the pair of O—C distances were restrained to within 0.01 Å of each other. The anisotropic temperature factors of the C atoms of the groups as well as those of the water molecule were tightly restrained to be nearly isotropic.

Omitted from the refinement were several reflections affected by the beamstop: (0 0 2), (0 1 0), (0 - 1 1), (1 - 1 1), (0 1 1), (0 1 2), (-1 1 0), (-1 1 1), (0 2 0), (0 0 1) and (0 0 3).

### Crystal data

**Table:**

[Fe(C_12_H_8_N_2_)_3_](C_9_H_5_N_4_O)_2_·0.5H_2_O	*Z* = 2
*M**_r_* = 975.81	*F*(000) = 1006
Triclinic, *P*1	*D*_x_ = 1.355 Mg m^−^^3^
Hall symbol: -P 1	Mo *K*α radiation, λ = 0.71073 Å
*a* = 9.3497 (3) Å	Cell parameters from 7674 reflections
*b* = 14.1736 (4) Å	θ = 2.6–23.7°
*c* = 18.6086 (6) Å	µ = 0.38 mm^−^^1^
α = 94.462 (2)°	*T* = 293 K
β = 96.562 (1)°	Prism, red
γ = 101.129 (1)°	0.55 × 0.35 × 0.15 mm
*V* = 2391.12 (13) Å^3^	

### Data collection

**Table:**

Bruker SMART APEX CCD diffractometer	10845 independent reflections
Radiation source: fine-focus sealed tube	6909 reflections with *I* > 2σ(*I*)
Graphite monochromator	*R*_int_ = 0.043
ω scans	θ_max_ = 27.5°, θ_min_ = 2.9°
Absorption correction: multi-scan (*SADABS*; Sheldrick, 1996)	*h* = −12→9
*T*_min_ = 0.820, *T*_max_ = 0.946	*k* = −18→18
38167 measured reflections	*l* = −23→24

### Refinement

**Table:**

Refinement on *F*^2^	Primary atom site location: structure-invariant direct methods
Least-squares matrix: full	Secondary atom site location: difference Fourier map
*R*[*F*^2^ > 2σ(*F*^2^)] = 0.049	Hydrogen site location: inferred from neighbouring sites
*wR*(*F*^2^) = 0.134	H-atom parameters constrained
*S* = 1.02	*w* = 1/[σ^2^(*F*_o_^2^) + (0.0594*P*)^2^ + 0.6022*P*] where *P* = (*F*_o_^2^ + 2*F*_c_^2^)/3
10845 reflections	(Δ/σ)_max_ = 0.001
661 parameters	Δρ_max_ = 0.36 e Å^−^^3^
40 restraints	Δρ_min_ = −0.43 e Å^−^^3^

### Fractional atomic coordinates and isotropic or equivalent isotropic displacement parameters (Å^2^)

**Table:**

	*x*	*y*	*z*	*U*_iso_*/*U*_eq_	Occ. (<1)
Fe1	0.50347 (3)	0.85774 (2)	0.224833 (17)	0.03344 (11)	
O1	0.3012 (2)	0.22872 (14)	0.20343 (10)	0.0616 (5)	
O2	1.14419 (19)	0.66090 (12)	0.39687 (10)	0.0537 (5)	
O1W	0.7463 (5)	0.5451 (3)	0.1682 (3)	0.0894 (15)	0.50
H11	0.6566	0.5202	0.1662	0.134*	0.50
H12	0.7908	0.5022	0.1546	0.134*	0.50
N1	0.5443 (2)	0.76256 (13)	0.29244 (10)	0.0361 (4)	
N2	0.39483 (19)	0.89919 (13)	0.30300 (10)	0.0376 (4)	
N3	0.4689 (2)	0.95925 (14)	0.16118 (10)	0.0380 (4)	
N4	0.67007 (19)	0.95977 (14)	0.27045 (10)	0.0368 (4)	
N5	0.6138 (2)	0.80877 (13)	0.15141 (10)	0.0367 (4)	
N6	0.3394 (2)	0.76187 (13)	0.17130 (10)	0.0370 (4)	
N7	0.4217 (3)	0.4827 (2)	0.16948 (15)	0.0812 (8)	
N8	−0.0519 (3)	0.4093 (2)	0.12339 (18)	0.0859 (9)	
N9	−0.1061 (3)	0.1837 (2)	0.02106 (15)	0.0761 (8)	
N10	0.0661 (3)	0.0069 (2)	0.17246 (17)	0.0878 (9)	
N11	0.9634 (3)	0.7897 (2)	0.28465 (15)	0.0779 (8)	
N12	0.8732 (3)	0.87480 (19)	0.49783 (14)	0.0709 (7)	
N13	0.8445 (4)	0.6721 (2)	0.58681 (16)	0.0889 (9)	
N14	1.2477 (4)	0.5698 (2)	0.57159 (18)	0.0987 (10)	
C1	0.4990 (2)	0.77928 (17)	0.35844 (12)	0.0394 (5)	
C2	0.6104 (3)	0.68844 (17)	0.28387 (14)	0.0456 (6)	
H2	0.6416	0.6750	0.2392	0.055*	
C3	0.6353 (3)	0.6299 (2)	0.33882 (16)	0.0576 (7)	
H3	0.6793	0.5774	0.3298	0.069*	
C4	0.5956 (3)	0.6491 (2)	0.40522 (16)	0.0592 (8)	
H4	0.6148	0.6115	0.4425	0.071*	
C5	0.5254 (3)	0.72648 (19)	0.41700 (13)	0.0495 (6)	
C6	0.4788 (3)	0.7556 (2)	0.48418 (15)	0.0650 (8)	
H6	0.4979	0.7226	0.5243	0.078*	
C7	0.4086 (3)	0.8287 (2)	0.49128 (14)	0.0643 (8)	
H7	0.3815	0.8459	0.5363	0.077*	
C8	0.3742 (3)	0.88111 (19)	0.43108 (14)	0.0499 (6)	
C9	0.2964 (3)	0.9560 (2)	0.43273 (16)	0.0605 (8)	
H9	0.2642	0.9766	0.4756	0.073*	
C10	0.2684 (3)	0.9981 (2)	0.37134 (17)	0.0613 (8)	
H10	0.2157	1.0474	0.3721	0.074*	
C11	0.3178 (3)	0.96841 (18)	0.30664 (15)	0.0489 (6)	
H11A	0.2960	0.9981	0.2651	0.059*	
C12	0.4207 (2)	0.85541 (17)	0.36471 (12)	0.0383 (5)	
C13	0.5731 (3)	1.04231 (17)	0.17762 (13)	0.0410 (6)	
C14	0.3676 (3)	0.9556 (2)	0.10430 (13)	0.0486 (6)	
H14	0.2952	0.8998	0.0924	0.058*	
C15	0.3654 (4)	1.0324 (2)	0.06164 (16)	0.0637 (8)	
H15	0.2933	1.0268	0.0219	0.076*	
C16	0.4689 (4)	1.1150 (2)	0.07842 (17)	0.0659 (8)	
H16	0.4680	1.1662	0.0501	0.079*	
C17	0.5771 (3)	1.1230 (2)	0.13850 (15)	0.0546 (7)	
C18	0.6900 (4)	1.2064 (2)	0.1631 (2)	0.0712 (9)	
H18	0.6951	1.2606	0.1377	0.085*	
C19	0.7891 (4)	1.2086 (2)	0.2221 (2)	0.0703 (9)	
H19	0.8596	1.2646	0.2372	0.084*	
C20	0.7877 (3)	1.12635 (19)	0.26187 (16)	0.0524 (7)	
C21	0.8876 (3)	1.1224 (2)	0.32361 (17)	0.0632 (8)	
H21	0.9611	1.1758	0.3415	0.076*	
C22	0.8755 (3)	1.0399 (2)	0.35668 (16)	0.0596 (7)	
H22	0.9398	1.0368	0.3980	0.071*	
C23	0.7672 (3)	0.96011 (19)	0.32887 (14)	0.0464 (6)	
H23	0.7619	0.9040	0.3521	0.056*	
C24	0.6805 (3)	1.04381 (17)	0.23827 (13)	0.0407 (6)	
C25	0.5282 (3)	0.73853 (16)	0.10219 (12)	0.0371 (5)	
C26	0.7553 (3)	0.83529 (19)	0.14283 (13)	0.0456 (6)	
H26	0.8160	0.8828	0.1760	0.055*	
C27	0.8151 (3)	0.7944 (2)	0.08606 (15)	0.0561 (7)	
H27	0.9139	0.8154	0.0815	0.067*	
C28	0.7299 (3)	0.7238 (2)	0.03722 (15)	0.0583 (8)	
H28	0.7702	0.6962	−0.0006	0.070*	
C29	0.5804 (3)	0.69269 (18)	0.04414 (13)	0.0463 (6)	
C30	0.4781 (4)	0.6204 (2)	−0.00332 (15)	0.0624 (8)	
H30	0.5106	0.5886	−0.0420	0.075*	
C31	0.3358 (4)	0.5970 (2)	0.00644 (15)	0.0609 (8)	
H31	0.2720	0.5501	−0.0260	0.073*	
C32	0.2801 (3)	0.64234 (17)	0.06550 (13)	0.0464 (6)	
C33	0.1346 (3)	0.6214 (2)	0.08017 (16)	0.0574 (7)	
H33	0.0648	0.5752	0.0499	0.069*	
C34	0.0957 (3)	0.6688 (2)	0.13894 (16)	0.0561 (7)	
H34	−0.0005	0.6542	0.1494	0.067*	
C35	0.1998 (3)	0.73890 (19)	0.18332 (14)	0.0478 (6)	
H35	0.1709	0.7710	0.2229	0.057*	
C36	0.3778 (3)	0.71246 (16)	0.11279 (12)	0.0368 (5)	
C37	0.4801 (14)	0.2478 (16)	0.3099 (11)	0.082 (3)	0.50
H37A	0.5131	0.2828	0.3570	0.123*	0.50
H37B	0.5590	0.2565	0.2807	0.123*	0.50
H37C	0.4493	0.1803	0.3150	0.123*	0.50
C38	0.3543 (10)	0.2847 (10)	0.2741 (5)	0.072 (2)	0.50
H38A	0.2758	0.2787	0.3044	0.086*	0.50
H38B	0.3854	0.3525	0.2675	0.086*	0.50
C37'	0.4354 (15)	0.2331 (16)	0.3176 (11)	0.082 (3)	0.50
H37D	0.5006	0.2716	0.3574	0.123*	0.50
H37E	0.4803	0.1826	0.2989	0.123*	0.50
H37F	0.3448	0.2052	0.3343	0.123*	0.50
C38'	0.4052 (10)	0.2956 (9)	0.2589 (5)	0.072 (2)	0.50
H38C	0.3616	0.3481	0.2767	0.086*	0.50
H38D	0.4946	0.3223	0.2395	0.086*	0.50
C39	0.3170 (3)	0.4245 (2)	0.16383 (15)	0.0592 (7)	
C40	0.1870 (3)	0.3516 (2)	0.15409 (14)	0.0501 (6)	
C41	0.0544 (3)	0.3820 (2)	0.13560 (16)	0.0588 (7)	
C42	0.1886 (3)	0.2547 (2)	0.16271 (13)	0.0504 (7)	
C43	0.0813 (3)	0.1764 (2)	0.13052 (14)	0.0511 (6)	
C44	−0.0228 (3)	0.1823 (2)	0.07024 (16)	0.0550 (7)	
C45	0.0744 (3)	0.0832 (2)	0.15443 (16)	0.0607 (8)	
C46	1.2014 (13)	0.5419 (14)	0.3149 (9)	0.095 (3)	0.50
H46A	1.2218	0.4780	0.3106	0.143*	0.50
H46B	1.2914	0.5886	0.3169	0.143*	0.50
H46C	1.1344	0.5492	0.2737	0.143*	0.50
C47	1.1335 (19)	0.5573 (5)	0.3836 (10)	0.069 (2)	0.50
H47A	1.0315	0.5236	0.3773	0.082*	0.50
H47B	1.1864	0.5336	0.4240	0.082*	0.50
C46'	1.1337 (13)	0.5284 (14)	0.3094 (9)	0.095 (3)	0.50
H46D	1.1242	0.4595	0.3026	0.143*	0.50
H46E	1.2107	0.5588	0.2838	0.143*	0.50
H46F	1.0426	0.5450	0.2909	0.143*	0.50
C47'	1.1709 (19)	0.5630 (5)	0.3900 (10)	0.069 (2)	0.50
H47C	1.1089	0.5218	0.4182	0.082*	0.50
H47D	1.2730	0.5627	0.4068	0.082*	0.50
C48	1.0607 (3)	0.69078 (17)	0.44425 (14)	0.0446 (6)	
C49	0.9870 (3)	0.76048 (18)	0.41913 (14)	0.0452 (6)	
C50	0.9760 (3)	0.7760 (2)	0.34445 (17)	0.0527 (7)	
C51	0.9244 (3)	0.8222 (2)	0.46418 (14)	0.0501 (6)	
C52	1.0539 (3)	0.65510 (19)	0.51216 (15)	0.0523 (7)	
C53	0.9382 (4)	0.6662 (2)	0.55343 (16)	0.0626 (8)	
C54	1.1624 (4)	0.6080 (2)	0.54415 (17)	0.0662 (8)	

### Atomic displacement parameters (Å^2^)

**Table:**

	*U*^11^	*U*^22^	*U*^33^	*U*^12^	*U*^13^	*U*^23^
Fe1	0.03130 (18)	0.0387 (2)	0.0314 (2)	0.00944 (14)	0.00527 (13)	0.00311 (14)
O1	0.0539 (11)	0.0735 (13)	0.0546 (12)	0.0231 (10)	−0.0069 (9)	−0.0160 (10)
O2	0.0536 (11)	0.0461 (11)	0.0661 (12)	0.0149 (9)	0.0178 (9)	0.0074 (9)
O1W	0.056 (2)	0.070 (3)	0.133 (4)	−0.001 (2)	0.027 (3)	−0.035 (3)
N1	0.0345 (10)	0.0384 (11)	0.0353 (11)	0.0076 (8)	0.0043 (8)	0.0028 (8)
N2	0.0326 (10)	0.0412 (11)	0.0382 (11)	0.0067 (8)	0.0059 (8)	0.0001 (9)
N3	0.0371 (10)	0.0451 (12)	0.0351 (11)	0.0146 (9)	0.0069 (9)	0.0054 (9)
N4	0.0328 (10)	0.0419 (11)	0.0369 (11)	0.0109 (8)	0.0061 (8)	0.0013 (9)
N5	0.0367 (10)	0.0427 (11)	0.0335 (11)	0.0122 (9)	0.0070 (9)	0.0077 (9)
N6	0.0378 (10)	0.0401 (11)	0.0335 (11)	0.0088 (9)	0.0052 (8)	0.0040 (9)
N7	0.0645 (18)	0.086 (2)	0.083 (2)	−0.0033 (16)	0.0072 (15)	−0.0035 (16)
N8	0.0693 (18)	0.0659 (18)	0.118 (2)	0.0124 (15)	−0.0099 (17)	0.0170 (16)
N9	0.0671 (17)	0.092 (2)	0.0589 (17)	0.0024 (15)	−0.0104 (14)	0.0039 (15)
N10	0.0750 (19)	0.076 (2)	0.109 (2)	0.0100 (16)	−0.0034 (17)	0.0215 (18)
N11	0.0790 (19)	0.111 (2)	0.0620 (18)	0.0474 (17)	0.0268 (15)	0.0255 (16)
N12	0.0793 (18)	0.0732 (18)	0.0631 (17)	0.0322 (15)	0.0048 (14)	−0.0110 (13)
N13	0.115 (3)	0.0733 (19)	0.086 (2)	0.0148 (17)	0.054 (2)	0.0074 (16)
N14	0.090 (2)	0.100 (2)	0.106 (2)	0.0214 (19)	−0.0108 (19)	0.042 (2)
C1	0.0386 (13)	0.0425 (14)	0.0332 (13)	−0.0002 (10)	0.0031 (10)	0.0027 (11)
C2	0.0463 (14)	0.0454 (15)	0.0472 (15)	0.0145 (12)	0.0049 (12)	0.0066 (12)
C3	0.0578 (17)	0.0507 (17)	0.067 (2)	0.0177 (13)	0.0038 (15)	0.0157 (14)
C4	0.0579 (17)	0.0590 (18)	0.0603 (19)	0.0076 (14)	−0.0012 (15)	0.0277 (15)
C5	0.0507 (15)	0.0564 (17)	0.0364 (15)	−0.0006 (13)	0.0002 (12)	0.0098 (12)
C6	0.068 (2)	0.081 (2)	0.0409 (17)	−0.0001 (17)	0.0043 (14)	0.0167 (15)
C7	0.073 (2)	0.082 (2)	0.0306 (15)	−0.0034 (17)	0.0154 (14)	0.0008 (14)
C8	0.0446 (14)	0.0558 (17)	0.0433 (15)	−0.0037 (12)	0.0127 (12)	−0.0078 (13)
C9	0.0578 (17)	0.069 (2)	0.0530 (18)	0.0037 (15)	0.0260 (14)	−0.0111 (15)
C10	0.0505 (16)	0.0605 (18)	0.076 (2)	0.0179 (14)	0.0230 (15)	−0.0115 (16)
C11	0.0425 (14)	0.0524 (16)	0.0554 (17)	0.0181 (12)	0.0105 (12)	0.0007 (13)
C12	0.0349 (12)	0.0448 (14)	0.0315 (13)	0.0001 (10)	0.0070 (10)	−0.0028 (10)
C13	0.0434 (13)	0.0407 (14)	0.0436 (14)	0.0130 (11)	0.0162 (11)	0.0074 (11)
C14	0.0487 (15)	0.0585 (17)	0.0403 (14)	0.0169 (13)	0.0009 (12)	0.0072 (12)
C15	0.072 (2)	0.079 (2)	0.0474 (17)	0.0319 (18)	0.0023 (15)	0.0184 (16)
C16	0.085 (2)	0.064 (2)	0.063 (2)	0.0322 (18)	0.0232 (17)	0.0317 (16)
C17	0.0641 (18)	0.0508 (17)	0.0581 (18)	0.0190 (14)	0.0254 (15)	0.0180 (14)
C18	0.081 (2)	0.0509 (19)	0.089 (3)	0.0115 (17)	0.035 (2)	0.0229 (17)
C19	0.067 (2)	0.0474 (18)	0.093 (3)	−0.0028 (15)	0.0266 (19)	−0.0027 (17)
C20	0.0481 (15)	0.0428 (16)	0.0644 (19)	0.0014 (12)	0.0178 (14)	−0.0023 (13)
C21	0.0471 (16)	0.0581 (19)	0.072 (2)	−0.0079 (14)	0.0056 (15)	−0.0180 (16)
C22	0.0418 (15)	0.073 (2)	0.0561 (18)	0.0065 (14)	−0.0024 (13)	−0.0122 (16)
C23	0.0372 (13)	0.0557 (16)	0.0447 (15)	0.0100 (12)	0.0016 (11)	0.0001 (12)
C24	0.0379 (13)	0.0397 (14)	0.0457 (15)	0.0088 (11)	0.0118 (11)	0.0000 (11)
C25	0.0473 (14)	0.0394 (13)	0.0286 (12)	0.0175 (11)	0.0043 (10)	0.0068 (10)
C26	0.0384 (13)	0.0591 (16)	0.0427 (15)	0.0139 (12)	0.0103 (11)	0.0083 (12)
C27	0.0499 (16)	0.078 (2)	0.0499 (17)	0.0255 (15)	0.0206 (14)	0.0153 (16)
C28	0.072 (2)	0.077 (2)	0.0416 (16)	0.0423 (17)	0.0249 (15)	0.0109 (15)
C29	0.0640 (17)	0.0507 (16)	0.0308 (13)	0.0268 (13)	0.0072 (12)	0.0060 (12)
C30	0.091 (2)	0.0595 (19)	0.0419 (16)	0.0319 (17)	0.0086 (16)	−0.0019 (14)
C31	0.085 (2)	0.0477 (17)	0.0438 (17)	0.0128 (15)	−0.0054 (15)	−0.0096 (13)
C32	0.0587 (16)	0.0385 (14)	0.0396 (15)	0.0099 (12)	−0.0043 (12)	0.0050 (12)
C33	0.0582 (18)	0.0459 (16)	0.0581 (19)	−0.0028 (13)	−0.0112 (14)	0.0044 (14)
C34	0.0400 (14)	0.0572 (18)	0.0651 (19)	−0.0030 (13)	0.0010 (13)	0.0110 (15)
C35	0.0374 (13)	0.0552 (16)	0.0497 (16)	0.0047 (12)	0.0085 (12)	0.0064 (13)
C36	0.0452 (13)	0.0351 (13)	0.0310 (13)	0.0117 (11)	0.0002 (10)	0.0067 (10)
C37	0.069 (7)	0.107 (6)	0.065 (4)	0.019 (6)	−0.006 (5)	−0.002 (3)
C38	0.085 (6)	0.089 (4)	0.046 (4)	0.040 (4)	−0.001 (3)	−0.012 (3)
C37'	0.069 (7)	0.107 (6)	0.065 (4)	0.019 (6)	−0.006 (5)	−0.002 (3)
C38'	0.085 (6)	0.089 (4)	0.046 (4)	0.040 (4)	−0.001 (3)	−0.012 (3)
C39	0.0559 (18)	0.070 (2)	0.0483 (17)	0.0075 (16)	0.0082 (14)	−0.0055 (14)
C40	0.0497 (15)	0.0563 (17)	0.0422 (15)	0.0085 (13)	0.0055 (12)	−0.0003 (13)
C41	0.0578 (18)	0.0516 (17)	0.0642 (19)	0.0052 (14)	0.0025 (15)	0.0112 (14)
C42	0.0437 (14)	0.0711 (19)	0.0377 (15)	0.0164 (14)	0.0074 (12)	−0.0015 (13)
C43	0.0476 (15)	0.0593 (18)	0.0468 (16)	0.0133 (13)	0.0048 (12)	0.0044 (13)
C44	0.0518 (16)	0.0635 (18)	0.0467 (17)	0.0045 (14)	0.0077 (14)	0.0029 (14)
C45	0.0505 (17)	0.072 (2)	0.0584 (19)	0.0134 (15)	0.0040 (14)	0.0028 (16)
C46	0.132 (9)	0.079 (5)	0.080 (3)	0.027 (7)	0.032 (7)	−0.003 (3)
C47	0.076 (7)	0.0497 (19)	0.088 (4)	0.022 (3)	0.029 (4)	0.0052 (19)
C46'	0.132 (9)	0.079 (5)	0.080 (3)	0.027 (7)	0.032 (7)	−0.003 (3)
C47'	0.076 (7)	0.0497 (19)	0.088 (4)	0.022 (3)	0.029 (4)	0.0052 (19)
C48	0.0407 (13)	0.0393 (14)	0.0518 (16)	0.0043 (11)	0.0081 (12)	−0.0003 (12)
C49	0.0419 (14)	0.0455 (15)	0.0476 (16)	0.0081 (11)	0.0085 (12)	−0.0007 (12)
C50	0.0445 (15)	0.0611 (18)	0.0580 (19)	0.0185 (13)	0.0149 (13)	0.0085 (15)
C51	0.0466 (15)	0.0532 (17)	0.0488 (16)	0.0120 (13)	−0.0002 (13)	0.0002 (13)
C52	0.0560 (16)	0.0477 (16)	0.0509 (17)	0.0064 (13)	0.0051 (13)	0.0030 (13)
C53	0.085 (2)	0.0438 (17)	0.0564 (19)	0.0035 (15)	0.0148 (17)	0.0034 (14)
C54	0.069 (2)	0.0602 (19)	0.066 (2)	0.0021 (16)	0.0033 (16)	0.0191 (16)

### Geometric parameters (Å, º)

**Table:**

Fe1—N6	1.9563 (19)	C19—C20	1.426 (4)
Fe1—N5	1.9654 (18)	C19—H19	0.9300
Fe1—N4	1.9686 (19)	C20—C24	1.390 (3)
Fe1—N1	1.9752 (18)	C20—C21	1.407 (4)
Fe1—N2	1.9819 (18)	C21—C22	1.355 (4)
Fe1—N3	1.9836 (18)	C21—H21	0.9300
O1—C42	1.353 (3)	C22—C23	1.385 (4)
O1—C38	1.463 (6)	C22—H22	0.9300
O1—C38'	1.473 (6)	C23—H23	0.9300
O2—C48	1.339 (3)	C25—C29	1.401 (3)
O2—C47	1.452 (6)	C25—C36	1.424 (3)
O2—C47'	1.455 (6)	C26—C27	1.389 (3)
O1W—H11	0.8400	C26—H26	0.9300
O1W—H12	0.8400	C27—C28	1.359 (4)
N1—C2	1.326 (3)	C27—H27	0.9300
N1—C1	1.362 (3)	C28—C29	1.405 (4)
N2—C11	1.326 (3)	C28—H28	0.9300
N2—C12	1.367 (3)	C29—C30	1.426 (4)
N3—C14	1.328 (3)	C30—C31	1.344 (4)
N3—C13	1.364 (3)	C30—H30	0.9300
N4—C23	1.334 (3)	C31—C32	1.429 (4)
N4—C24	1.366 (3)	C31—H31	0.9300
N5—C26	1.336 (3)	C32—C36	1.388 (3)
N5—C25	1.361 (3)	C32—C33	1.398 (4)
N6—C35	1.332 (3)	C33—C34	1.360 (4)
N6—C36	1.368 (3)	C33—H33	0.9300
N7—C39	1.140 (4)	C34—C35	1.390 (4)
N8—C41	1.140 (4)	C34—H34	0.9300
N9—C44	1.136 (3)	C35—H35	0.9300
N10—C45	1.149 (4)	C37—C38	1.487 (9)
N11—C50	1.141 (3)	C37—H37A	0.9600
N12—C51	1.145 (3)	C37—H37B	0.9600
N13—C53	1.142 (4)	C37—H37C	0.9600
N14—C54	1.143 (4)	C38—H38A	0.9700
C1—C5	1.395 (3)	C38—H38B	0.9700
C1—C12	1.421 (3)	C37'—C38'	1.495 (9)
C2—C3	1.393 (3)	C37'—H37D	0.9600
C2—H2	0.9300	C37'—H37E	0.9600
C3—C4	1.353 (4)	C37'—H37F	0.9600
C3—H3	0.9300	C38'—H38C	0.9700
C4—C5	1.398 (4)	C38'—H38D	0.9700
C4—H4	0.9300	C39—C40	1.419 (4)
C5—C6	1.427 (4)	C40—C42	1.398 (4)
C6—C7	1.334 (4)	C40—C41	1.404 (4)
C6—H6	0.9300	C42—C43	1.388 (4)
C7—C8	1.432 (4)	C43—C44	1.417 (4)
C7—H7	0.9300	C43—C45	1.418 (4)
C8—C9	1.398 (4)	C46—C47	1.509 (9)
C8—C12	1.400 (3)	C46—H46A	0.9600
C9—C10	1.352 (4)	C46—H46B	0.9600
C9—H9	0.9300	C46—H46C	0.9600
C10—C11	1.401 (4)	C47—H47A	0.9700
C10—H10	0.9300	C47—H47B	0.9700
C11—H11A	0.9300	C46'—C47'	1.521 (9)
C13—C17	1.399 (3)	C46'—H46D	0.9600
C13—C24	1.418 (3)	C46'—H46E	0.9600
C14—C15	1.398 (4)	C46'—H46F	0.9600
C14—H14	0.9300	C47'—H47C	0.9700
C15—C16	1.357 (4)	C47'—H47D	0.9700
C15—H15	0.9300	C48—C49	1.389 (3)
C16—C17	1.401 (4)	C48—C52	1.401 (4)
C16—H16	0.9300	C49—C51	1.416 (4)
C17—C18	1.430 (4)	C49—C50	1.419 (4)
C18—C19	1.348 (4)	C52—C54	1.419 (4)
C18—H18	0.9300	C52—C53	1.422 (4)
			
N6—Fe1—N5	83.02 (8)	N5—C25—C29	123.8 (2)
N6—Fe1—N4	174.79 (7)	N5—C25—C36	115.8 (2)
N5—Fe1—N4	94.35 (8)	C29—C25—C36	120.3 (2)
N6—Fe1—N1	90.18 (7)	N5—C26—C27	122.3 (3)
N5—Fe1—N1	93.45 (7)	N5—C26—H26	118.8
N4—Fe1—N1	94.48 (8)	C27—C26—H26	118.8
N6—Fe1—N2	96.56 (8)	C28—C27—C26	120.3 (3)
N5—Fe1—N2	175.80 (7)	C28—C27—H27	119.9
N4—Fe1—N2	86.38 (7)	C26—C27—H27	119.9
N1—Fe1—N2	82.36 (7)	C27—C28—C29	119.7 (2)
N6—Fe1—N3	92.90 (8)	C27—C28—H28	120.2
N5—Fe1—N3	88.01 (7)	C29—C28—H28	120.2
N4—Fe1—N3	82.50 (8)	C25—C29—C28	116.5 (2)
N1—Fe1—N3	176.74 (8)	C25—C29—C30	117.7 (3)
N2—Fe1—N3	96.18 (7)	C28—C29—C30	125.7 (2)
C42—O1—C38	116.7 (6)	C31—C30—C29	121.6 (3)
C42—O1—C38'	123.4 (6)	C31—C30—H30	119.2
C48—O2—C47	116.9 (7)	C29—C30—H30	119.2
C48—O2—C47'	123.0 (7)	C30—C31—C32	121.6 (3)
H11—O1W—H12	108.7	C30—C31—H31	119.2
C2—N1—C1	116.7 (2)	C32—C31—H31	119.2
C2—N1—Fe1	130.32 (16)	C36—C32—C33	116.9 (2)
C1—N1—Fe1	112.96 (15)	C36—C32—C31	117.8 (3)
C11—N2—C12	117.2 (2)	C33—C32—C31	125.3 (3)
C11—N2—Fe1	130.11 (17)	C34—C33—C32	119.7 (3)
C12—N2—Fe1	112.15 (14)	C34—C33—H33	120.2
C14—N3—C13	117.4 (2)	C32—C33—H33	120.2
C14—N3—Fe1	129.94 (18)	C33—C34—C35	120.0 (3)
C13—N3—Fe1	112.52 (15)	C33—C34—H34	120.0
C23—N4—C24	116.6 (2)	C35—C34—H34	120.0
C23—N4—Fe1	129.93 (17)	N6—C35—C34	122.5 (2)
C24—N4—Fe1	113.38 (15)	N6—C35—H35	118.7
C26—N5—C25	117.4 (2)	C34—C35—H35	118.7
C26—N5—Fe1	129.83 (17)	N6—C36—C32	124.0 (2)
C25—N5—Fe1	112.78 (15)	N6—C36—C25	115.2 (2)
C35—N6—C36	116.9 (2)	C32—C36—C25	120.8 (2)
C35—N6—Fe1	129.92 (17)	C38—C37—H37A	109.5
C36—N6—Fe1	113.17 (15)	C38—C37—H37B	109.5
N1—C1—C5	123.7 (2)	H37A—C37—H37B	109.5
N1—C1—C12	115.5 (2)	C38—C37—H37C	109.5
C5—C1—C12	120.8 (2)	H37A—C37—H37C	109.5
N1—C2—C3	122.9 (2)	H37B—C37—H37C	109.5
N1—C2—H2	118.5	O1—C38—C37	109.1 (12)
C3—C2—H2	118.5	O1—C38—H38A	109.9
C4—C3—C2	120.2 (3)	C37—C38—H38A	109.9
C4—C3—H3	119.9	O1—C38—H38B	109.9
C2—C3—H3	119.9	C37—C38—H38B	109.9
C3—C4—C5	119.1 (2)	H38A—C38—H38B	108.3
C3—C4—H4	120.5	C38'—C37'—H37D	109.5
C5—C4—H4	120.5	C38'—C37'—H37E	109.5
C1—C5—C4	117.3 (2)	H37D—C37'—H37E	109.5
C1—C5—C6	117.5 (3)	C38'—C37'—H37F	109.5
C4—C5—C6	125.2 (3)	H37D—C37'—H37F	109.5
C7—C6—C5	122.2 (3)	H37E—C37'—H37F	109.5
C7—C6—H6	118.9	O1—C38'—C37'	103.5 (12)
C5—C6—H6	118.9	O1—C38'—H38C	111.1
C6—C7—C8	121.4 (3)	C37'—C38'—H38C	111.1
C6—C7—H7	119.3	O1—C38'—H38D	111.1
C8—C7—H7	119.3	C37'—C38'—H38D	111.1
C9—C8—C12	116.8 (2)	H38C—C38'—H38D	109.0
C9—C8—C7	125.3 (3)	N7—C39—C40	178.0 (3)
C12—C8—C7	117.9 (2)	C42—C40—C41	121.1 (2)
C10—C9—C8	119.3 (2)	C42—C40—C39	122.6 (2)
C10—C9—H9	120.4	C41—C40—C39	116.3 (3)
C8—C9—H9	120.4	N8—C41—C40	177.0 (4)
C9—C10—C11	120.9 (3)	O1—C42—C43	113.2 (2)
C9—C10—H10	119.6	O1—C42—C40	121.5 (2)
C11—C10—H10	119.6	C43—C42—C40	125.2 (2)
N2—C11—C10	121.9 (3)	C42—C43—C44	123.1 (3)
N2—C11—H11A	119.1	C42—C43—C45	121.1 (2)
C10—C11—H11A	119.1	C44—C43—C45	115.7 (3)
N2—C12—C8	123.9 (2)	N9—C44—C43	177.6 (3)
N2—C12—C1	115.9 (2)	N10—C45—C43	178.3 (3)
C8—C12—C1	120.2 (2)	C47—C46—H46A	109.5
N3—C13—C17	123.5 (2)	C47—C46—H46B	109.5
N3—C13—C24	116.1 (2)	H46A—C46—H46B	109.5
C17—C13—C24	120.3 (2)	C47—C46—H46C	109.5
N3—C14—C15	122.6 (3)	H46A—C46—H46C	109.5
N3—C14—H14	118.7	H46B—C46—H46C	109.5
C15—C14—H14	118.7	O2—C47—C46	106.0 (12)
C16—C15—C14	119.8 (3)	O2—C47—H47A	110.5
C16—C15—H15	120.1	C46—C47—H47A	110.5
C14—C15—H15	120.1	O2—C47—H47B	110.5
C15—C16—C17	119.8 (3)	C46—C47—H47B	110.5
C15—C16—H16	120.1	H47A—C47—H47B	108.7
C17—C16—H16	120.1	C47'—C46'—H46D	109.5
C13—C17—C16	116.8 (3)	C47'—C46'—H46E	109.5
C13—C17—C18	117.6 (3)	H46D—C46'—H46E	109.5
C16—C17—C18	125.5 (3)	C47'—C46'—H46F	109.5
C19—C18—C17	121.8 (3)	H46D—C46'—H46F	109.5
C19—C18—H18	119.1	H46E—C46'—H46F	109.5
C17—C18—H18	119.1	O2—C47'—C46'	105.4 (12)
C18—C19—C20	121.1 (3)	O2—C47'—H47C	110.7
C18—C19—H19	119.4	C46'—C47'—H47C	110.7
C20—C19—H19	119.4	O2—C47'—H47D	110.7
C24—C20—C21	117.2 (3)	C46'—C47'—H47D	110.7
C24—C20—C19	118.1 (3)	H47C—C47'—H47D	108.8
C21—C20—C19	124.7 (3)	O2—C48—C49	112.9 (2)
C22—C21—C20	119.3 (3)	O2—C48—C52	121.9 (2)
C22—C21—H21	120.3	C49—C48—C52	125.2 (2)
C20—C21—H21	120.3	C48—C49—C51	124.2 (2)
C21—C22—C23	119.9 (3)	C48—C49—C50	120.1 (2)
C21—C22—H22	120.0	C51—C49—C50	115.7 (2)
C23—C22—H22	120.0	N11—C50—C49	177.9 (3)
N4—C23—C22	123.3 (3)	N12—C51—C49	176.6 (3)
N4—C23—H23	118.4	C48—C52—C54	122.4 (3)
C22—C23—H23	118.4	C48—C52—C53	121.2 (2)
N4—C24—C20	123.7 (2)	C54—C52—C53	116.4 (3)
N4—C24—C13	115.4 (2)	N13—C53—C52	177.9 (3)
C20—C24—C13	120.9 (2)	N14—C54—C52	178.1 (4)
			
N6—Fe1—N1—C2	77.2 (2)	N3—C13—C17—C18	178.1 (2)
N5—Fe1—N1—C2	−5.8 (2)	C24—C13—C17—C18	−1.6 (4)
N4—Fe1—N1—C2	−100.5 (2)	C15—C16—C17—C13	1.2 (4)
N2—Fe1—N1—C2	173.8 (2)	C15—C16—C17—C18	−178.5 (3)
N6—Fe1—N1—C1	−104.61 (16)	C13—C17—C18—C19	−0.5 (4)
N5—Fe1—N1—C1	172.37 (16)	C16—C17—C18—C19	179.2 (3)
N4—Fe1—N1—C1	77.72 (16)	C17—C18—C19—C20	1.5 (5)
N2—Fe1—N1—C1	−8.02 (15)	C18—C19—C20—C24	−0.3 (4)
N6—Fe1—N2—C11	−89.7 (2)	C18—C19—C20—C21	179.6 (3)
N4—Fe1—N2—C11	86.0 (2)	C24—C20—C21—C22	0.1 (4)
N1—Fe1—N2—C11	−179.0 (2)	C19—C20—C21—C22	−179.8 (3)
N3—Fe1—N2—C11	3.9 (2)	C20—C21—C22—C23	1.1 (4)
N6—Fe1—N2—C12	98.77 (15)	C24—N4—C23—C22	−0.6 (3)
N4—Fe1—N2—C12	−85.53 (15)	Fe1—N4—C23—C22	−176.88 (18)
N1—Fe1—N2—C12	9.48 (15)	C21—C22—C23—N4	−0.9 (4)
N3—Fe1—N2—C12	−167.58 (15)	C23—N4—C24—C20	1.9 (3)
N6—Fe1—N3—C14	0.1 (2)	Fe1—N4—C24—C20	178.83 (19)
N5—Fe1—N3—C14	83.0 (2)	C23—N4—C24—C13	−178.1 (2)
N4—Fe1—N3—C14	177.6 (2)	Fe1—N4—C24—C13	−1.2 (2)
N2—Fe1—N3—C14	−96.8 (2)	C21—C20—C24—N4	−1.7 (4)
N6—Fe1—N3—C13	−175.51 (15)	C19—C20—C24—N4	178.2 (2)
N5—Fe1—N3—C13	−92.61 (15)	C21—C20—C24—C13	178.4 (2)
N4—Fe1—N3—C13	2.05 (15)	C19—C20—C24—C13	−1.7 (4)
N2—Fe1—N3—C13	87.56 (15)	N3—C13—C24—N4	3.1 (3)
N5—Fe1—N4—C23	−96.6 (2)	C17—C13—C24—N4	−177.2 (2)
N1—Fe1—N4—C23	−2.8 (2)	N3—C13—C24—C20	−177.0 (2)
N2—Fe1—N4—C23	79.2 (2)	C17—C13—C24—C20	2.7 (3)
N3—Fe1—N4—C23	176.0 (2)	C26—N5—C25—C29	0.5 (3)
N5—Fe1—N4—C24	86.99 (15)	Fe1—N5—C25—C29	179.39 (17)
N1—Fe1—N4—C24	−179.20 (15)	C26—N5—C25—C36	−179.68 (19)
N2—Fe1—N4—C24	−97.16 (15)	Fe1—N5—C25—C36	−0.8 (2)
N3—Fe1—N4—C24	−0.43 (15)	C25—N5—C26—C27	0.5 (3)
N6—Fe1—N5—C26	179.6 (2)	Fe1—N5—C26—C27	−178.19 (18)
N4—Fe1—N5—C26	4.2 (2)	N5—C26—C27—C28	−0.9 (4)
N1—Fe1—N5—C26	−90.6 (2)	C26—C27—C28—C29	0.4 (4)
N3—Fe1—N5—C26	86.5 (2)	N5—C25—C29—C28	−1.0 (3)
N6—Fe1—N5—C25	0.94 (14)	C36—C25—C29—C28	179.2 (2)
N4—Fe1—N5—C25	−174.54 (14)	N5—C25—C29—C30	−179.9 (2)
N1—Fe1—N5—C25	90.70 (15)	C36—C25—C29—C30	0.3 (3)
N3—Fe1—N5—C25	−92.22 (15)	C27—C28—C29—C25	0.5 (4)
N5—Fe1—N6—C35	179.3 (2)	C27—C28—C29—C30	179.3 (2)
N1—Fe1—N6—C35	85.9 (2)	C25—C29—C30—C31	0.6 (4)
N2—Fe1—N6—C35	3.5 (2)	C28—C29—C30—C31	−178.2 (3)
N5—Fe1—N6—C36	−0.92 (14)	C29—C30—C31—C32	−0.9 (4)
N1—Fe1—N6—C36	−94.38 (15)	C30—C31—C32—C36	0.4 (4)
N2—Fe1—N6—C36	−176.71 (14)	C30—C31—C32—C33	−179.0 (3)
N3—Fe1—N6—C36	86.72 (15)	C36—C32—C33—C34	−0.4 (4)
C2—N1—C1—C5	3.4 (3)	C31—C32—C33—C34	179.0 (2)
Fe1—N1—C1—C5	−175.04 (18)	C32—C33—C34—C35	1.1 (4)
C2—N1—C1—C12	−176.4 (2)	C36—N6—C35—C34	−0.5 (3)
Fe1—N1—C1—C12	5.2 (3)	Fe1—N6—C35—C34	179.29 (18)
C1—N1—C2—C3	−0.6 (4)	C33—C34—C35—N6	−0.7 (4)
Fe1—N1—C2—C3	177.50 (19)	C35—N6—C36—C32	1.2 (3)
N1—C2—C3—C4	−2.1 (4)	Fe1—N6—C36—C32	−178.56 (17)
C2—C3—C4—C5	2.0 (4)	C35—N6—C36—C25	−179.46 (19)
N1—C1—C5—C4	−3.4 (4)	Fe1—N6—C36—C25	0.7 (2)
C12—C1—C5—C4	176.4 (2)	C33—C32—C36—N6	−0.8 (3)
N1—C1—C5—C6	176.5 (2)	C31—C32—C36—N6	179.7 (2)
C12—C1—C5—C6	−3.7 (4)	C33—C32—C36—C25	179.9 (2)
C3—C4—C5—C1	0.5 (4)	C31—C32—C36—C25	0.5 (3)
C3—C4—C5—C6	−179.4 (3)	N5—C25—C36—N6	0.0 (3)
C1—C5—C6—C7	1.9 (4)	C29—C25—C36—N6	179.86 (19)
C4—C5—C6—C7	−178.2 (3)	N5—C25—C36—C32	179.37 (19)
C5—C6—C7—C8	1.0 (5)	C29—C25—C36—C32	−0.8 (3)
C6—C7—C8—C9	177.4 (3)	C42—O1—C38—C37	−178.4 (7)
C6—C7—C8—C12	−2.0 (4)	C42—O1—C38'—C37'	143.5 (8)
C12—C8—C9—C10	1.0 (4)	C38—O1—C42—C43	−136.6 (6)
C7—C8—C9—C10	−178.5 (3)	C38'—O1—C42—C43	−162.0 (5)
C8—C9—C10—C11	−0.7 (4)	C38—O1—C42—C40	44.9 (6)
C12—N2—C11—C10	1.9 (4)	C38'—O1—C42—C40	19.5 (6)
Fe1—N2—C11—C10	−169.21 (19)	C41—C40—C42—O1	−156.7 (3)
C9—C10—C11—N2	−0.8 (4)	C39—C40—C42—O1	22.8 (4)
C11—N2—C12—C8	−1.7 (3)	C41—C40—C42—C43	24.9 (4)
Fe1—N2—C12—C8	171.01 (19)	C39—C40—C42—C43	−155.6 (3)
C11—N2—C12—C1	177.9 (2)	O1—C42—C43—C44	−161.2 (2)
Fe1—N2—C12—C1	−9.4 (2)	C40—C42—C43—C44	17.4 (4)
C9—C8—C12—N2	0.2 (4)	O1—C42—C43—C45	15.6 (3)
C7—C8—C12—N2	179.7 (2)	C40—C42—C43—C45	−165.9 (3)
C9—C8—C12—C1	−179.3 (2)	C48—O2—C47—C46	164.3 (7)
C7—C8—C12—C1	0.2 (3)	C47'—O2—C47—C46	−76 (6)
N1—C1—C12—N2	2.9 (3)	C48—O2—C47'—C46'	128.5 (8)
C5—C1—C12—N2	−176.9 (2)	C47—O2—C48—C49	−133.8 (8)
N1—C1—C12—C8	−177.5 (2)	C47'—O2—C48—C49	−148.0 (8)
C5—C1—C12—C8	2.7 (3)	C47—O2—C48—C52	47.5 (9)
C14—N3—C13—C17	0.8 (3)	C47'—O2—C48—C52	33.3 (9)
Fe1—N3—C13—C17	176.96 (18)	O2—C48—C49—C51	−162.4 (2)
C14—N3—C13—C24	−179.5 (2)	C52—C48—C49—C51	16.3 (4)
Fe1—N3—C13—C24	−3.3 (2)	O2—C48—C49—C50	15.0 (3)
C13—N3—C14—C15	0.4 (3)	C52—C48—C49—C50	−166.3 (2)
Fe1—N3—C14—C15	−174.98 (19)	O2—C48—C52—C54	18.5 (4)
N3—C14—C15—C16	−0.7 (4)	C49—C48—C52—C54	−160.1 (3)
C14—C15—C16—C17	−0.1 (4)	O2—C48—C52—C53	−162.8 (2)
N3—C13—C17—C16	−1.6 (4)	C49—C48—C52—C53	18.7 (4)
C24—C13—C17—C16	178.7 (2)		

### Hydrogen-bond geometry (Å, º)

**Table:**

*D*—H···*A*	*D*—H	H···*A*	*D*···*A*	*D*—H···*A*
O1w—H11···N7	0.84	2.17	2.996 (5)	169
O1w—H12···N8^i^	0.84	2.25	3.078 (5)	169

Symmetry code: (i) *x*+1, *y*, *z*.
